# On the Uterine Supporter

**Published:** 1842-03-26

**Authors:** Wm. Harris


### MEDICAL EXAMINER.

NEW SERIES.

No. 13.] PHILADELPHIA, MARCH 26, 1842. [Vol. I.

### *On the Uterine Supporter.* By Wm. Harris, M. D.

A lecture upon prolapsus uteri, which I delivered at the Medical Institute, in the Spring of 1839, was published in the May number of the Medical Examiner of the same year; and since that time the advantages and disadvantages of the *uterine supporter* have been freely discussed and its usefulness extensively tested.

Like every new improvement, the supporter met with formidable opposition, even from gentlemen of elevated standing in the profession, but it slowly crept into favour, and its superiority is now almost univeisally acknowledged. More than a hundred medical gentlemen in this city, among whom are Professors in each of the four Medical Schools, have tried it on their patients afflicted with prolapsus uteri, and are testifying to its great advantages over every other apparatus employed by the profession; and more than a thousand females either radically cured, or materially benefited, are earnestly recommending it to their fellow sufferers.

With whom the idea of an external uterine supporter originated is uncertain; I apprehend, however, that the honour is due to the late Doctor Dewees, whose labours have contributed so much to enlarge the boundaries of obstetrical science, and to adorn the history of the earliest and most celebrated medical institution of our country, that he well deserves the appellation of the *Father of American Obstetrics.*

While attending one of his lectures, in the University of Pennsylvania, upon prolapsus uteri, I heard him remark that the pessary occasioned, in some females, so much uterine and vaginal irritation, and consequent leucorrhœa, that they were obliged to discontinue the use of it, and that he hoped an instrument would some day be invented that would restore the displaced organ, by making external pressure upon the abdomen and perineum. This remark, perhaps through some medical students, reached the ear of Dr. Hull of New-York, and induced him to bring forward the first uterine supporter that ever was presented to the public. Subsequently a great variety of supporters were invented, by different persons, in various parts of the United States, the best of which by a Mrs. Betts, a well educated and accomplished lady, a native of London, now residing in the city of Philadelphia.* She invented the apparatus for her own case, which, under the care of Professor Jackson, was conducted to such a successful issue that she was induced afterwards to offer the instrument to the medical profession. In the last three years, I have tried her supporter in my own practice, and in consultation, on upwards of fifty females, with great success, in some producing radical cures, and in others great comfort, so that I feel constrained to give it my cordial recommendation.

The time required to perform a radical cure by the supporter, varies from three to eigtheen months, in most cases one year.

*South-west corner of Third and Tammany streets.

A lady of this city, thirty-eight years of age, afflicted with procidentia uteri, consulted me in her case about three years since. She remarked that she had worn pessaries of various kinds for a length of time, all of which produced great irritation and leucorrhœa, and that she ascribed to this cause an abortion that she had had a few weeks before. She said this misfortune had caused her deep sorrow, having but one child, and that she was determined never again to submit to the introduction of a pessary. Besides the depression of her spirits, her general health was very feeble, and the only exercise she attempted to take was to walk down three or four steps, from her chamber to the dining room, to eat her meals.

I procured for her a uterine supporter, and after it was applied and properly adjusted, she felt so comfortable and strong, that, it being Sunday morning, she decided to walk with her husband to church, and, returning home but little fatigued, she went again in the afternoon. By the use of the supporter, chalybeates, and exercise in the open air, on foot, and in a carriage, she improved so rapidly that after one year, her health was established and her procidentia cured; and now she is able to attend to all her usual avocations without the encumbrance of a supporter. Nevertheless, as a prophylactic measure, when she expects to walk for three or four hours, shopping, or making morning calls, she puts it on. She is now one of the most healthy and efficient ladies in Philadelphia.

Another lady, about six weeks after her confinement, fell down stairs, by which a prolapsus uteri was produced. Being sent for immediately, I ordered her to bed, where she was confined three days, at the end of which time Mrs. Betts applied a supporter, which in three months effected a radical cure. A number of other cases, under my care, were radically cured, after a great variety of pessaries had been tried in vain, by the most skilful physicians.

For my success in the treatment of these cases I am much indebted to the assistance of the inventor of the apparatus. I now cause her to apply it to all my patients labouring under the complaint, unless objections are made, so that she can alter and adjust it to each female's particular shape. Without this precaution, the instrument will not give so comfortable a support to the patient, and acure will not be so rapidly or effectually accomplished.

Again: the perineal pad, when first applied, sometimes produces great irritation of the skin and unpleasant heat in the soft parts; when this is the case, I cause the pad to be removed for a few days, and, instead of it, to have applied under the perineal strap a soft folded napkin; and this substitution of the napkin for the pad is always necessary during the menstrual period, or, what sometimes answers equally well, to roll the napkin around the pad to prevent it from being soiled. Females, indeed, that are very particular, wear a piece of old linen or muslin around the pad always, that it may be kept perfectly clean. Mrs. Betts's supporter has been introduced into practice in New York, and in various other parts of the United States, and the testimony in its favour, from all quarters, is unequivocal.

I have found the supporter to be, also, a most effectual preventive of *habitual* abortion. Some females of leuco-phlegmatic temperament, great nervous irritability and feeble health, are subject to habitual abortion, which ordinarily takes place during the early months of pregnancy. At the return of every menstrual period, regular periodical uterine pains come on, threatening a premature delivery. Such cases, under proper management, may be conducted safely to the full period, and the accoucheur have the pleasure of presenting to the anxious mother a living child, as the reward of her repeated sufferings.

As soon as the threatening pains come on, direct the patient to go immediately to bed, order dry cups to be applied over the sacrum, sometimes scarifying two, or four, so as to take away from two to four ounces of blood, according to circumstances, administering at the same time an anodyne enema, consisting of two ounces of flaxseed tea, or of a thin solution of starch, and from forty to a hundred drops of laudanum according to the urgency of the symptoms, and the capacity of the patient to bear narcotics. The foot-posts of the bedstead should be raised, at the same time, about six inches, by placing blocks of wood under them, so that the womb may be thrown upwards and points of irritation, thereby, be removed. This treatment, in a few hours, will put a stop to the threatening symptoms, but the patient must not be allowed to rise from her bed under two or three days, as the erect posture is apt to produce a relapse. Before she rises from her bed, a supporter should be carefully applied, which will give her great comfort, and enable her with safety to attend to her usual avocations.

Some authors recommend that patients, under such circumstances, should be kept in bed throughout the whole period of gestation; but such practice cannot be too much condemned, as in almost every instance it injures or destroys the female's health. Pregnant females that suffer from prolapsus uteri during the three first months of gestation, but at no other period, have also derived great comfort from wearing the supporter.

For a description of this apparatus, and the manner in which it is applied, I refer the reader to the 329th page of the second volume of the Medical Examiner. Every medical gentleman, however, from a glance at the apparatus, would at once comprehend the manner in which it should be applied. Mrs. Betts's last improvement, which she calls her *laced supporter*, is decidedly the best.

Notwithstanding the success that has attended the use of the supporter in Philadelphia, there are still a few medical gentlemen, who occupy an enviable position in the profession, that resist the introduction of this apparatus into their respective circles of practice. They object to its use because, as they allege, the pressure by the broad pad or belt over the hypogastric region must force the intestines downwards against the pelvic viscera, and thereby increase, instead of removing the disease. If this be true it is a valid objection, and the supporter ought to be laid aside. Let the facts decide. The axis of the pelvis is not parallel with the axis of the body, but strikes off from it at an angle of forty-five degrees. The centre of gravity being therefore in the direction of the axis of the body, the weight of the intestines must fall upon the symphysis pubis and upon the lower part of the parietes of the abdomen, especially if the female has borne children, and is consequently a little corpulent. Now it must be admitted, that if the pressure by the supporter were backwards and downwards, the intestines would be crowded upon the fundus uteri, and the displacement be consequently increased; but as the force which it exerts is upwards and backwards, exactly in the direction of the plane of the superior strait of the pelvis, or at right angles with its axis, I contend that it completely removes the weight of the intestines from the pelvic viscera, and that the uterus has consequently a tendency to rise to its natural position through the contractile power of its ligaments.

This objection then being, as I apprehend, completely removed, I proceed to examine another, which is, that the *perineal pad*, supported by the strap that passes between the limbs, does not raise the uterus sufficiently high to restore it to its primitive situation, and that a radical cure can therefore, never be effected.

As those gentlemen, who condemn the supporter, maintain that the pessary is better calculated to restore the displacement under discussion, I shall consider this opinion first.

Pessaries are made of various materials, and of different shapes, but the flat circular pessary, made of glass or of silver gilt, is the kind most in use. This pessary is about half an inch in thickness, and two inches in diameter; in introducing and adjusting which, it is recommended that it be placed in the vagina upon its edge, with the convex surface towards the rectum and parallel with it, the upper edge in the cul de sac at the upper end of the vagina, and behind the posterior lip of the os uteri, and the lower edge resting upon the perineum or floor of the pelvis, near the point of the os coccygis. It is maintained that the pessary thus adjusted will raise the uterus its whole breadth, nearly two inches, and there sustain it until the ligaments that support the uterus have time to contract and recover their primative tonicity, and a radical cure thus be accomplished. This is very plausible in theory, but difficult to reduce to practice.

If we commence at the posterior part of the superior strait of the pelvis, and pass along the hollow of the sacrum and coccyx and the floor of the pelvis up to the top of the symphysis pubis, we shall describe an arch amounting to nearly a semicircle. Now it is known that a weight capable of sliding upon a curved surface will not rest until it reaches the lowest position; consequently, if a pessary be placed in the vagina parallel with the rectum, while the patient is lying upon her back, it will remain in that position as long as she retains the recumbent posture; but as soon as she assumes the erect attitude, the pessary will slide down and rest with its convex surface parallel with the perineum, and now the thickness of the pessary, half an inch, is all that interposes between the os uteri and floor of the pelvis. The uterus is therefore only raised half an inch higher in the vagina than it was before the pessary was introduced, and if instead of the flat circular, the oblong or oval ring pessary were used, after it slides down, the os and cervix uteri would pass through it, rest upon the perineum, and therefore not be elevated at all.

Besides, if the pessary is found to answer effectually the purpose for which it is designed, why such an interminable change in its shape and dimensions! Now if it can be demonstrated that the *perineal pad and strap* of the supporter is capable of raising the uterus more than half an inch, and retaining it there permanently, as much will be accomplished by the supporter as can be done by the pessary, and the patient have the advantage of using an apparatus that is less irritating and less offensive to her sense of delicacy.

To prove this will not be difficult. The distance from the point of the os coccygis to the arch of the pubis is four inches and a half, which is filled up by very elastic soft parts that extend in the antero-posterior direction, not in a straight line, but curved downwards, so that the centre of the convexity projects at least one inch below a straight line. Now it requires no great stretch of the imagination to suppose that the convexity of the perineum could be pressed up by the pad to a straight line; nor is it difficult to conceive that by gradually shortening the perineal straps, the pressure might in time be so increased as to raise these parts one inch above the straight line, thereby raising the uterus two inches, which is more than can be accomplished by the pessary, even if it could be kept upon its edge and parallel with the rectum.

Such are the views which I entertain with regard to the use of the supporter in cases of prolapsed womb; and I have the satisfaction to know that I hold these opinions in common with a large majority of the medical profession, in and around Philadelphia.

